# The wizard behind the curtain: programmers as providers

**DOI:** 10.1186/s13010-016-0038-0

**Published:** 2016-08-04

**Authors:** Mark A. Graber, Olivia Bailey

**Affiliations:** 1 Department of Emergency Medicine, University of Iowa Carver College of Medicine, 200 Hawkins Drive, 1008 JCP, Iowa City, IA 52242 USA; 2 Department of Family Medicine, University of Iowa Carver College of Medicine, Iowa City, USA

## Abstract

It is almost universally accepted that traditional provider-patient relationships should be governed, at least in part, by the ethical principles set forth by Beauchamp and Childress (Beauchamp and Childress, Principles of biomedical ethics, 1979). These principles include autonomy, beneficence, non-maleficence and justice (Beauchamp and Childress, Principles of biomedical ethics, 1979). Recently, however, the nature of medial practice has changed. The pervasive presence of computer technology in medicine raises interesting ethical questions. In this paper we argue that some software designers should be considered health care providers and thus be subject the ethical principles incumbent upon “traditional” providers. We argue that these ethical responsibilities should be applied explicitly rather than as a passive, implicit, set of guidelines.

## Introduction

One of the fundamental raison-d’etres of bioethics is to protect the patient from providers who have the power to make diagnostic and treatment decisions. While in the past the providers’ power to harm (and benefit) in a medical context has been limited to traditional “healthcare” roles such as literally seeing a patient in the office, technology has changed the scope of what can be considered a provider-patient relationship. Two obvious examples of this are telemedicine and email; there is no longer any need for the patient and the provider to be in the same place nor even to interact synchronously. A less obvious example, we will argue, is code written for devices such as pacemakers and ventilators and code written into an “expert system”. In this paper we will examine the relationship between those who design medically related software and patients. It is our contention that at least in some cases a provider-patient relationship is established between those who design software and the patients whose diagnoses and treatments are dependent on that software.

We have to accomplish several goals in order to establish our case. First, we will show that decision support algorithms directly affect patient diagnosis and treatment. Software that has no effect on patients obviously can cause no benefit or harm. Second, we will show that decision support computer algorithms differ in some fundamental way from the “decision support” information in books and that this difference is enough to establish a new form of provider-patient relationship. Lastly, we will show that there is a reason to believe that software designers have some responsibility to the patient beyond that dictated by business ethics. *If* there is an ethical responsibility that mirrors that of a traditional provider, *then* we can infer a provider-patient relationship of some sort.

## Methods

No data was collected for this paper. The paper develops an ethical framework for considering programmers as providers and is entirely a thought piece.

### The players and the extent of our claim

It is important to first establish exactly whom we are talking about. When we use the term software designer, programmer and similar terms, we are talking about the person or people who have developed the algorithms for product control (such as pacemaker programming) or who have influenced the decision making process for expert systems. We are not including actual coders in our definition except to the extent that coding decisions play some role in the algorithm or expert system’s “thought processes.” When we use the term “software”, we stipulate that it refers only to decision support algorithms/computer-aided diagnosis/expert systems and not to electronic books, etc. We will make the distinction between these two categories clear further on in the paper.

It is also important to state up front that we are not claiming, nor are we trying to establish, that the provider-patient relationship for a software designer is the same as that of a traditional health care provider and her patient. However, we will argue that there are ethical duties from a bioethical perspective incumbent on the software designer as a provider. In any single case, the strength of these duties will depend on the degree to which decision-making algorithms or computerized protocols influence patient diagnosis or treatment.

### Why ask this question?

Why have this discussion? Because this is not a theoretical exercise. We already have medical devices that reflect the decision making of the software designer which have direct patient impact. One example of this is pacemakers. Take, for example, a 2013 study published in the Journal of the American Medical Association (JAMA) [2]. This study looked at the effect of pacemaker programming on the diagnosis and treatment of arrhythmias. It found that if the pacemaker was programmed to allow a longer period of arrhythmia detection before delivering a shock, fewer inappropriate shocks were delivered. While this particular study did not show a difference in mortality between the long and short detection time groups, this is likely because it only lasted for 1 year. In fact, prior studies have shown that allowing a longer time for arrhythmia detection before delivering a shock improves mortality [3]. So, the diagnostic and treatment algorithms designed for these pacemakers have a direct effect on patient outcome. This outcome is a direct reflection of the algorithm designer’s thought process and programming. A second illustrative example is the case of ventilator control software. Here, the programmer is no different from the physical provider at the bedside who orders changes to ventilator settings based on patient parameters. Even though this is done “automatically” based on programming, the software reflects the same type of thinking/decision making as that of a bedside provider. *In both of these cases, despite the physical absence of the programmer, the programmer-patient interaction is no less real from a consequentialist perspective*. In fact, there has been at least one Class I recall of ventilators based on faulty software [4]. A third example is found in computer-aided diagnosis. A 2013 study published in the Annals of Internal Medicine found that computer aided detection in screening mammography leads to more false-positive mammograms as well as more diagnoses of ductal carcinoma in situ [5]. The software is influencing decision making as evidenced by more biopsies performed in the computer-aided diagnosis group versus those suggested by standard, unaided, mammography readers. Presumably, this also leads to more complications, etc. in women who have had computer aided mammography. This is not a benign outcome; we already over-diagnose breast cancer leading to unnecessary interventions [6]. Finding more in situ disease will inevitably lead to more interventions of questionable benefit. So, not only does the software affect decision-making but, at least in this case, may cause more harm than good.

We have shown that programmers can have a direct effect on patient outcomes. But the importance of this discussion goes beyond the current scope of medical software; this discussion is also important to the future of medicine. The age of decision-making expert systems acting on their own is no longer speculative. IBM is developing “Watson,” a computer dedicated to medical diagnosis (Kelly, 2014 [7]). In fact, it has been claimed that, “…..something like Watson will soon be the world’s best diagnostician—whether machine or human (Kelly, 2014 [7]).” Given this claim, it behooves us to determine the moral standing of the computer and those behind it.

### Setting the stage

To this point we have shown that software influences patient care. But does this lead to a provider-patient relationship? To initiate this discussion, a thought experiment is in order. Let’s look at an example taken from the annals of science fiction, Star Trek to be exact. Dr. McCoy is dependent to a large degree on his “medical tricorder.” In fact, about the only medical advice that Dr. McCoy can give without a medical tricorder is, “He’s dead, Jim.” (and half of the time he is wrong….). The “medical tricorder” is essentially acting as a consultant *in absentia* giving advice based on programmed algorithms. The only difference between the medical tricorder and a consultant is the proximity of the individual giving advice to the patient. In the case of a live consultant, the consultant is generally at the bedside. *In the case of the tricorder, the consultant’s “thinking” about the case is coded by the programmer*. The same type of information is collected and digested as would be in an “in person” consult before an opinion is offered based on the algorithm developed by the consultant. But does the software really reflect the programmer’s thought process? It seems that the answer is “yes”. As noted by an expert in the philosophy of artificial intelligence (AI), “What we [are] actually doing when we code is describing the world from our perspective. Whatever the assumptions and biases we have in ourselves are very likely to replicated in [that] code” [11].

The diagnoses and treatments recommended by these instruments are clearly dependent on the expert system design. If the algorithms are wrong, the diagnosis is wrong. So, there has to be *some* degree of moral responsibility for the software designer because errors in algorithms and logic can result in patient harm and wasted resources.

One obvious objection to this thesis is that Dr. McCoy can ignore the advice given by the medical tricorder and that the medical tricorder is only a consultant. If Dr. McCoy judges that it is in the patient’s best interest to ignore the tricorder he can do so. However, this does not differentiate the medical tricorder from a flesh-and-blood consultant. The primary provider is free to take a consultant’s advice or not. That the primary provider can ignore a consultant’s advice does not diminish consultant’s responsibility for diagnostic accuracy. So the fact that the provider can ignore the advice of the expert system does not change the expert system’s moral status.

To this point we have shown that a “medical tricorder” wielded by a physician (or other provider) plays a role that is analogous to that of a consultant. Now lets remove Dr. McCoy from the scenario. We now have a device capable of making diagnostic and treatment decisions when wielded, for example, by a lay-person. The responsibility for the outcomes here is clear: a programmed device is making diagnostic and treatment decisions that reflect the thinking of the algorithm designer(s) who evaluated the evidence and made recommendations. So, essentially, it puts the programmer in the position of provider, albeit while physically removed from the patient.

There is a fundamental challenge to this position: In the case of true artificial intelligence an autonomous “machine” (with “machine” in quotes because the status of such a machine as “person” has not been established) will be responsible for itself. This is an interesting question. To the extent that the development of the “machine’s” artificial intelligence is inevitably the result of its original programming (as noted above) [11], there is still some responsibility on the part of the programmer.

### Books and computer expert systems are different in kind

The reader may be wondering how Dr. McCoy “consulting” an expert system is different from Dr. McCoy consulting a book. It can be argued that expert systems are no different from offering information in a book: Book based information helps providers make decisions. This criticism fails, however. First, in the case of autonomously functioning software (e.g. the pacemaker or tricorder without Dr. McCoy discussed above), there is no human intermediary. The software, and therefore the programmer, is the final arbiter of the diagnosis and treatment.

Second, expert systems either autonomously make decisions or synthesize existing data *based on an individuals symptoms, labs,* etc. to come to a likely diagnosis. This is clearly a different function than simply providing factual information such as that in a book and allowing the provider to synthesize the information of any individual patient into a diagnosis and treatment on her own.

One might argue that in the case of non-autonomous devices the provider is still making the final decision. While this is true, it does not change the fundamental difference between expert systems and books. A book can be “read like a book” and “is an open book”. These English idioms point out that information in a book is (ideally!) clearly laid out available to the reader. *In contrast, expert systems are opaque; the decision making process is generally not open to the end user*. This would certainly be the case with autonomous expert systems. When we trust the software, we simply trust that the contribution of the software to our diagnostic accuracy is correct.

Our final argument here is an empirical one. The legal status of medical expert systems or software control systems is different from that of electronic books. For example, the FDA classifies decision-making software and “electronic books” differently [8]. One of the fundamental questions asked by the FDA is when determining if a software program is subject to regulation or not is, “Is it simply an electronic version of some existing library source material?” [8] If the answer is “yes”, the software need not be reviewed by the FDA. If “no”, the software is put into a different category from that of an electronic medical reference. Hence it may be subject to review by the FDA.

### Expert systems, programmers, and the provider-patient relationship

We have established that diagnostic and treatment algorithms can have a direct affect on patient outcome whether or not there is a human intermediary. But this still does not establish a provider-patient relationship. One can argue that this scenario is the same as a curbside consult which does not establish a provider-patient relationship [9]. In the case of a curbside consult, the patient has not been examined and there is no contractural relationship between the patient and the provider [9, 10].

We have three reasons for thinking that some sort of provider-patient relationship is in play here and that this is different than a “curbside consult”. The first is that there is economic activity going on. The software designer is offering herself as an expert *and hoping to profit from her design and expertise*. In the case of a curbside consult there is no economic activity extant and therefore no contract, either implicit or explicit. In the case of expert systems or medical device programming, the designer is offering his opinion as an expert and is clearly “being paid” for this expertise, albeit not in a traditional model [12]. We aren’t arguing that the provider-patient relationship is the same as the traditional one that we currently recognize. But it is recognizable as a tacit contract being offered by the programmer: a service is being offered, is applied to an individual patient using that patient’s clinical data, and the programmer is being paid.

Of course, the presence of economic activity in and of itself does not establish a provider-patient relationship. Take, for example, drug companies and medications. Here there is economic activity related to patient care: The drug company is being paid for a medication. However, the drug company is not using the parameters of any particular patient nor making decisions about the care of any particular patient. Drugs are more analogous to books: there is economic activity going on (one is buying an electronic book or a drug) but there is no interaction, face-to-face or via software, with any particular patient’s individual data leading to the offering of a specific diagnosis or treatment.

Our second reason for thinking that a provider-patient relationship is in play has to do with part of the *raison d’etre* of bioethics: to protect the patient. We have shown above that there is a direct effect on patient outcomes based on software design. Were there no outcome implications, there would be no issue. Arguing backwards, this relationship meets at least the spirit of what can be considered the purview of bioethics. The ability to harm (or benefit) a patient *by making decisions based on an individual’s particular case* puts the software designer in the de facto position of a provider.

A third, and perhaps the most important reason, has to do with patient autonomy. Generally, a patient will go to a provider because he trusts the provider’s judgment. The patient may meticulously research a provider online (and elsewhere) before deciding whom to trust. Whether this in anyway protects the patient’s interests is an important question. But for our purposes, this is irrelevant. The point is that regardless of the astuteness of the decision, the patient is making an autonomous choice about whose judgment to trust. In the case of expert systems the patient is unknowingly giving up some degree of autonomy to an expert system without her knowledge. *It is not the solely the provider who is making diagnostic and treatment decisions but rather a hybrid computer-provider dyad*.

There is a possible solution to this. If the algorithm/expert system was completely open, the provider would be able to understand and review each step taken by the expert system in making a treatment recommendation; the provider could review the decision making process to see if it is one that he trusts. In this case the provider is “asking” the consultant about his thinking and questioning the decision making process. This will certainly lessen the responsibility of the programmer but will not eliminate it. Again it is analogous to a corporeal consultant: Kudos (or blame) are meted out based on the degree of involvement of the consultant. For example, if a consultant suggests one thing but the primary care provider takes a different course, the adverse outcome will be attributed to the primary care provider. If both agree and there is a bad outcome, this will be ascribed to both providers.

Another possible solution is to let the patient know ahead of time that software will be contributing to the decision making process. This way, the patient will at least be informed of the process that leads to the final diagnosis and treatment plan and understand that there is input beyond that of the practitioner. She can then decide if this is a partnership she wants to enter in to.

### Principles of medical ethics

So which of Beauchamp and Childress' prinicples of medical ethics do we believe should apply in the case of a software designer [1]? Clearly, it is not our typical understanding of justice. Software will treat each individual the same, regardless of economic status, etc. This is with the caveat that all individuals have access to the particular device, something not under control of the programmer.

We would suggest that autonomy applies at least to some degree. The patient is unknowingly ceding some autonomy to the software; the software is directly affecting the patient’s diagnosis and treatment without his knowledge. Normally, we hold forth “informed consent” as the ideal yardstick by which we measure patient autonomy [13]. Problems with informed consent aside [12], it is unreasonable to expect a software designer to be responsible for getting consent in each case. As noted above, a discussion with the patient about computer added diagnosis and treatment might be in order. At the very least it is important to let the patient know that the recommendations are the result of a collaboration between the software and the corporeal provider.

We also believe that the principles of beneficence and non-maleficence apply. Beyond simple business ethics, software designers, as a type of provider, have a responsibility to act in a fiduciary manner and to not cause harm to patients. As noted above, in the case of some control algorithms and computer-aided diagnosis, this standard is not being met.

### The current regimen

The FDA already has a role in supervising some of these devices. For purposes of the FDA, “Medical Device Software” includes software “intended for use in the diagnosis of disease or other conditions, or in the cure, mitigation, treatment, or prevention of disease…. in man [sic]….” [8] One may consider this oversight adequate protection for the patient. However, changes to approved devices thought to be incremental innovations may be approved without requiring the manufacturers to submit any clinical data. Between 1979 and 2012, the FDA approved 77 original premarket approval process applications (including clinical data) for cardiac electronic implantable devices while approving 5829 supplement applications (without clinical data) during the same time period [14]. The programming of the original device has usually been changed and without post-approval surveillance. This is important; if the algorithm driving the device’s “decisions” has changed, so may the patient outcome. While it is possible that each iteration of a program leads to improvements, this is not a given. Anyone who has worked with a computer has experienced crashes and suffering performance after installing new software. So the patients’ interests are not necessarily protected by the current regimen.

It can be argued that software designers are already subject to a code of ethics. However, this code generally applies to business practices rather than the programmer-as-a provider. The increasingly central role of programmers in the provision of patient care demands explicit acknowledgement of, and training in, bioethics to directly remind the programmer of her responsibility in patient care.

## Conclusion

We have shown that computer and other programming based technology affects patients’ diagnoses and, by extension, treatment. As such, the programmer of such software can be considered a category of provider for the reasons noted above.
